# Fabrication of a three-dimensional micro/nanocarbon structure with sub-10 nm carbon fiber arrays based on the nanoforming and pyrolysis of polyacrylonitrile-based jet fibers

**DOI:** 10.1038/s41378-023-00604-1

**Published:** 2023-10-16

**Authors:** Jufeng Deng, Chong Liu, Dian Song, Marc Madou

**Affiliations:** 1 Key Laboratory of Advanced Manufacturing Technology of the Ministry of Education, Guizhou University, Guiyang, China; 2 School of Mechanical Engineering, Dalian University of Technology, Dalian, China; 3 Chemical and Biomolecular Engineering, University of California, Irvine, USA; 4 Mechanical and Aerospace Engineering, University of California, Irvine, USA; 5 School of Engineering and Science, Tecnologico de Monterrey, Monterrey, Mexico

**Keywords:** Nanowires, Nanowires

## Abstract

To produce a three-dimensional micro/nanocarbon structure, a manufacturing design technique for sub-10 nm carbon fiber arrays on three-dimensional carbon micropillars has been developed; the method involves initiating electrostatic jetting, forming submicron-to-nanoscale PAN-based fibers, and maximizing the shrinkage from polyacrylonitrile (PAN)-based fibers to carbon fibers. Nanoforming and nanodepositing methods for polyacrylonitrile-based jet fibers as precursors of carbon fibers are proposed for the processing design of electrostatic jet initiation and for the forming design of submicron-to-nanoscale PAN-based fibers by establishing and analyzing mathematical models that include the diameter and tensile stress values of jet fibers and the electric field intensity values on the surfaces of carbon micropillars. In accordance with these methods, an array of jet fibers with diameters of ~80 nm is experimentally formed based on the thinning of the electrospinning fluid on top of a dispensing needle, the poking of drum into an electrospinning droplet, and the controlling of the needle–drum distance. When converting thin PAN-based jet fibers to carbon fibers, a pyrolysis method consisting of the suspension of jet nanofibers between carbon micropillars, the bond between the fibers and the surface of the carbon micropillar, and the control of micropillar spacing, stabilization temperature, and carbonation rate is presented to maximize the shrinkage from PAN-based fibers to carbon fibers and to form sub-10 nm carbon fiber arrays between three-dimensional carbon micropillars. The manufacturing design of a three-dimensional micro/nanocarbon structure can produce thin PAN-based jet nanofibers and nanofiber arrays aligned on micropillar surfaces, obtain shrinkage levels reaching 96% and incorporate sub-10 nm carbon fibers into three-dimensional carbon micropillars; these actions provide new research opportunities for correlated three-dimensional micro/nanocarbon structures that have not previously been technically possible.

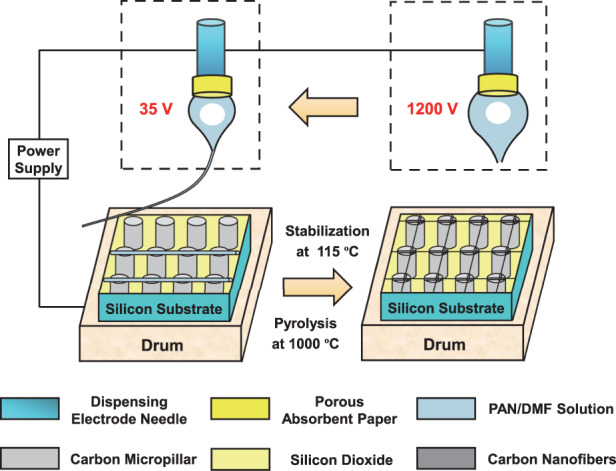

## Introduction

Three-dimensional (3D) carbon micropillars have been fabricated to emphasize the effects of scale and specific surface area on performance for energy storage systems^1^, highly sensitive detection devices^2^, lithium-ion capacitors^3^, battery systems^4^ and general electrochemistry applications^5,6^. Carbon nanofiber arrays are expected to be arrayed on 3D carbon micropillars (3DCMPs) to highlight the effects of nanoscale and highly specific surface areas on performance, enabling the development of multifunctional devices with excellent performance levels. Typical methods represented by chemical vapor deposition have been conducted to fabricate such carbon nanofiber grown on the surfaces of different bulk substrates^7–9^. however, it is difficult for these carbon fibers to be deposited onto the surface of microstructures on the bulk substrates in an orderly manner. The forming and carbonization of fibers obtained through electrospinning from polymeric precursors, such as polyacrylonitrile^10^ and photoresist (SU-8)^11^, has become an effective alternative to arraying carbon fibers on the surfaces of carbon micropillars. The resulting diameters of carbon fibers in arrays have increased from the nanometer scale to the submicron scale, ranging from 100 nm to 1000 nm.

Intensive efforts to reduce the diameters of carbon fibers in arrays to the nanometer scale must be made; these efforts include forming thin polymer fibers in near-field electrospinning and increasing the percentage of shrinkage in the conversion of polymer fibers to carbon fibers during pyrolysis for carbon fiber-based nanostructures on micropillars. By using low-voltage near-field electrospinning, Bisht et al.^12^ demonstrated that the diameters of polyethylene oxide fibers can be decreased to 16.2 nm at a low voltage of 200 V by introducing a very high local electric field with a glass microprobe tip that is 1–3 μm in tip diameter. However, polyethylene oxide fibers cannot be converted to carbon fibers by carbonization; for pyrolyzable polymers such as photoresist (SU-8), this method is accompanied by an increase in the diameter of those polymer fibers to the submicron scale, obtaining carbon nanowires with diameters of ~180 nm. In addition, when high shrinkage occurs during fiber conversion, existing methods are adopted by weakening the limiting effect of the deposited surface on fiber radial shrinkage^13^, mechanically stretching the fiber^14^, and thermally inducing shrinkage^15^. However, research related to these methods has been difficult to devote to further increasing shrinkage to 90%, making the resulting carbon fibers remain at the micron scale. Therefore, a key impediment for the fabrication of carbon nanofiber arrays on micropillars is the lack of methods for fabricating thin pyrolyzable polymer fibers in near-field electrospinning and maximizing the radial shrinkage of the fibers during stabilization and pyrolysis.

In this work, nanoforming and nanodeposition methods for polyacrylonitrile (PAN)-based fibers in near-field electrospinning are proposed for PAN-based nanofiber arrays on 3DCMPs by establishing mathematical models of diameter-based control and fiber-based deposition, analyzing these models and performing experiments. The subsequent pyrolysis method of these PAN fibers in the carbonization procedure is introduced by a protocol and a control method to increase the radial shrinkage of fibers to a relatively great extent. The nanoforming, nanodepositing and pyrolysis methods enable carbon fibers to be arrayed onto carbon micropillars; additionally, their diameters are reduced to less than 10 nm, achieving a 3D micro/nanocarbon structure with sub-10 nm carbon fiber arrays and 3D carbon micropillars.

## Mathematical modeling and theoretical analysis

### Diameter-based control for PAN fibers in near-field electrospinning

**Fig. 1:**
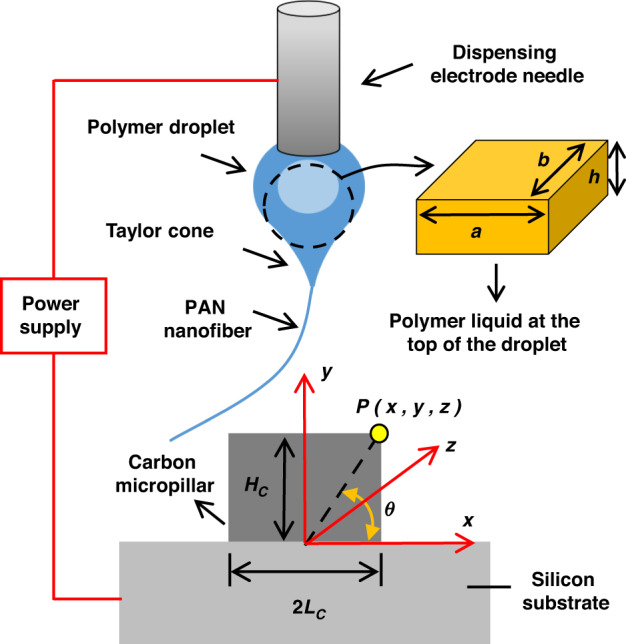
Schematic of low-voltage near-field electrospinning. The evolution of electrospinning solutions to jet fibers and their deposition onto carbon microcolumns by virtue of electrostatics

The process of low-voltage near-field electrospinning in Fig. 1 was initiated by applying the adsorption threshold voltage of the electrospinning solution and introducing an artificial instability at the droplet–air interface^12,16^. The adsorption threshold voltage became sufficiently small to reduce the diameter of the polymer fiber. By assuming that the main forces acting on the electrospun liquid are electrostatic and elastic forces, the governing equations for the applied voltage *V* related to the adsorption threshold voltage are determined to be as follows:

1 $$\left\{\begin{array}{l}{F}_{ele}-{F}_{K}=m\frac{{d}^{2}y}{d{t}^{2}},\,{F}_{ele}=\frac{\varepsilon A{V}^{2}}{2{({g}_{0}-y)}^{2}}\\ {F}_{K}=Ky,\,K=\frac{Eb{h}^{3}}{2{a}^{3}}\end{array}\right.$$

where *F*_ele_ is an electrostatic force, *F*_K_ is an elastic force, *m* is the mass of the electrospinning liquid, *y* is the axial displacement of the electrospinning fluid in Fig. 1, *Ɛ* is the dielectric constant, *A* is the area of the electrospinning fluid faced with the collector, *g*_0_ is the axial distance between the electrospinning fluid and the collector, *K* is the elastic coefficient, *E* is the modulus of elasticity, *b* is the width of the electrospinning fluid in Fig. 1h is the thickness of the electrospinning solution in Fig. 1a is the length of the electrospinning solution in Fig. 1e is the strength of the electrostatic field.

Considering the critical condition of the adsorption threshold voltage for adsorption of electrospinning liquid onto the surface of the collector, *d*^2^*y*/*dt*^2^ = 0 should be satisfied. As *d*^2^*y*/*dt*^2^ is substituted into Eq. (1), the moving displacement can be given as follows:

2 $$\left\{\begin{array}{l}{y}_{1}={y}_{2}=\frac{1}{3}{g}_{0},\,{y}_{3}=\frac{4}{3}{g}_{0},\,{y}_{4}=\frac{2}{3}{g}_{0}(1-\,\cos \frac{\theta }{3})\\ {y}_{5}=\frac{2{g}_{0}}{3}(1+\,\cos \frac{\theta +\pi }{3}),\,{y}_{6}=\frac{2{g}_{0}}{3}(1+\,\cos \frac{\theta -\pi }{3})\\ \theta =\arccos T,\,T=1-\frac{108\varepsilon A{V}^{2}}{16K{{g}_{0}}^{3}}\end{array}\right.$$

where *y*_1_, *y*_2_, *y*_3_, *y*_4_, *y*_5_, and *y*_6_ are the moving displacements when the electrostatic force is equal to the elastic force. In Equation (2), *y*_3_ > *g*_0_ and *y*_6_ > *g*_0_ are not consistent with the actual situation and do not have to be considered. The remaining displacements in Eq. (2) and *d*^2^*y*/*dt*^2^ = 0 are substituted into Eq. (1), and the resulting voltage can be obtained as follows:

3 $$\left\{\begin{array}{l}{V}_{P}=\sqrt{\frac{8K{{g}_{0}}^{3}}{27\varepsilon A}}\,\,({\rm{y}}={y}_{1}={y}_{2})\\ {V}_{R}=\sqrt{\frac{2Ky{({g}_{0}-y)}^{2}}{\varepsilon A}}\,\,({\rm{y}}={y}_{4}\,or\,y={y}_{5})\end{array}\right.$$

*V*_P_ > *V*_R_ in Eq. (3) indicates that *V*_R_ corresponds to the adsorption threshold voltage of the electrospinning solution. According to the energy conservation law for the adsorption of electrospinning liquid onto the surface of the collector at the adsorption threshold voltage, the critical equation is derived as follows:

4 $${\int }_{0}^{{y}_{4}}\frac{\varepsilon A{V}^{2}}{2{({g}_{0}-y)}^{2}}dy-{\int }_{0}^{{y}_{4}}Kydy={\int }_{{y}_{4}}^{{y}_{5}}Kydy-{\int }_{{y}_{4}}^{{y}_{5}}\frac{\varepsilon A{V}^{2}}{2{({g}_{0}-y)}^{2}}dy\;\;(V={V}_{R})$$

By combining Eq. (3) with Eq. (4), the final expression for the adsorption threshold voltage of electrospinning solution *V*_R_ is obtained as follows:

5 $$\left\{\begin{array}{l}{V}_{R}=\sqrt{\frac{{g}_{0}K{y}_{5}({g}_{0}-{y}_{5})}{\varepsilon A}},\,K=\frac{Eb{h}^{3}}{2{a}^{3}}\\ {y}_{5}=\frac{2{g}_{0}}{3}(1+\,\cos \frac{\theta +\pi }{3}),\,\theta =\arccos T\\ T=1-\frac{108\varepsilon A{{V}_{R}}^{2}}{16K{{g}_{0}}^{3}}\end{array}\right.$$

At the adsorption threshold voltage, artificial instability at the droplet–air interface is introduced to overcome the interfacial surface tension, initiating the polymer jet and forming jet fibers. The cross-sectional radius of the jet fiber *r* is given as follows^17^:

6 $$r=InverseFunction\left\{\left[-\frac{{{E}_{1}}^{2}{{K}_{1}}^{2}In{\rm{y}}}{8{I}^{3}}+\frac{{{E}_{1}}^{2}{{K}_{1}}^{2}In(-2I+{E}_{1}{K}_{1}{y}^{2})}{16{I}^{3}}+\frac{1}{8I{y}^{4}}+\frac{{E}_{1}{K}_{1}}{8{I}^{2}{y}^{2}}\right]\left[\frac{{E}_{1}y}{2{Q}^{3}\sqrt{\beta }}+{C}_{1}\right]\right\}$$

where *E*_1_ is the strength of the electric field, *K*_1_ is the electric conductivity of the electrospinning solution, *I* is the total current in the electrified jet, *Q* is the volume flow rate, *β* is the dimensionless conductivity of the electrospinning fluid, and *C*_1_ is a constant.

In accordance with the relationship between the electric field strength and the voltage, the strength of the electric field can be shown as *E*_1_ = *V*_R_/(*g*_0_-*y*). By combining *E*_1_ = *V*_R_/(*g*_0_-*y*) with Eq. (5) and Eq. (6), the diameters of polymer fibers in near-field electrospinning are obtained as follows:

7 $$\left\{\begin{array}{l}r=InverseFunction \left\{\left[-\frac{{{E}_{1}}^{2}{{K}_{1}}^{2}In{\rm{y}}}{8{I}^{3}}+\frac{{{E}_{1}}^{2}{{K}_{1}}^{2}In(-2I+{E}_{1}{K}_{1}{y}^{2})}{16{I}^{3}}+\frac{1}{8I{y}^{4}}+\frac{{E}_{1}{K}_{1}}{8{I}^{2}{y}^{2}}\right]\left[\frac{{E}_{1}y}{2{Q}^{3}\sqrt{\beta }}+{C}_{1}\right]\right\}\\ {E}_{1}=\frac{{V}_{R}}{{{\rm{g}}}_{0}-y},\,{V}_{R}=\sqrt{\frac{{g}_{0}K{y}_{5}({g}_{0}-{y}_{5})}{\varepsilon A}},\,K=\frac{Eb{h}^{3}}{2{a}^{3}},\,{y}_{5}=\frac{2{g}_{0}}{3}(1+\,\cos \frac{\theta +\pi }{3})\\ \theta =\arccos T,\,T=1-\frac{108\varepsilon A{{V}_{thr}}^{2}}{16K{{g}_{0}}^{3}}\end{array}\right.$$

Investigation into the effects of the factors in Eq. (7) on the diameter of the polymer fiber reveals a decreasing trend of voltage versus diameter. As in a previous study, this trend explains well the reason that low voltage in near-field electrospinning enables the thicknesses of polymer fibers to reach the nanometer scale^12^. Furthermore, in accordance with the relationship between the thickness and the adsorption threshold voltage of the electrospinning solution in Eq. (7), the thinning of *h* in Fig. 1 allows the low voltage for the adsorption of the electrospun liquid at the top of the droplet onto the surface of the collector during artificial instability. Upon forming jet fibers after artificial instability, a decrease in the thickness of the electrospinning solution at the top of the droplet can effectively reduce the diameter of the jet fiber at a low volume flow rate, which is discovered by analyzing Eq. (7).

### Fiber-based deposition in near-field electrospinning

Various deficiencies during fiber deposition, such as curving and beading, emerge as a result of the relative velocity between the velocity of the fiber and the linear velocity of the collector^18^. The velocity of the fiber dependent on the velocity of the jet from the nozzle is given as follows^17^:

8 $$v=\frac{Q}{{{\rm{r}}}^{2}},\,r=InverseFunction \left\{\left[\begin{array}{c}-\frac{{{E}_{1}}^{2}{{K}_{1}}^{2}In{\rm{y}}}{8{I}^{3}}+\frac{1}{8I{y}^{4}}+\frac{{E}_{1}{K}_{1}}{8{I}^{2}{y}^{2}}\\ +\frac{{{E}_{1}}^{2}{{K}_{1}}^{2}In(-2I+{E}_{1}{K}_{1}{y}^{2})}{16{I}^{3}}\end{array}\right]\left[\frac{{E}_{1}y}{2{Q}^{3}\sqrt{\beta }}+{C}_{1}\right]\right\}$$

where *v* is the velocity of the fiber. By using Eq. (8), the tensile stress of the fiber that is derived from this relative velocity can be obtained as follows:

9 $$\left\{\begin{array}{l}F=\frac{{{\rm{m}}}_{1}({v}_{1}-\frac{Q}{{r}^{2}})}{\pi {r}^{2}\varDelta t},\,r=InverseFunction \left\{\left[\begin{array}{c}-\frac{{{E}_{1}}^{2}{{K}_{1}}^{2}In{\rm{y}}}{8{I}^{3}}+\frac{1}{8I{y}^{4}}+\frac{{E}_{1}{K}_{1}}{8{I}^{2}{y}^{2}}\\ +\frac{{{E}_{1}}^{2}{{K}_{1}}^{2}In(-2I+{E}_{1}{K}_{1}{y}^{2})}{16{I}^{3}}\end{array}\right]\left[\frac{{E}_{1}y}{2{Q}^{3}\sqrt{\beta }}+{C}_{1}\right]\right\}\\ {E}_{1}=\frac{{V}_{R}}{{{\rm{g}}}_{0}-y},\,{V}_{R}=\sqrt{\frac{{g}_{0}K{y}_{5}({g}_{0}-{y}_{5})}{\varepsilon A}},\,K=\frac{Eb{h}^{3}}{2{a}^{3}},\,{y}_{5}=\frac{2{g}_{0}}{3}(1+\,\cos \frac{\theta +\pi }{3})\\ \theta =\arccos T,\,T=1-\frac{108\varepsilon A{{V}_{thr}}^{2}}{16K{{g}_{0}}^{3}}\end{array}\right.$$

where *F* is the tensile stress of the jet fiber, *v*_1_ is the linear velocity of the collector, *m*_1_ is the mass of the jet fiber, and Δ*t* is the time at which the jet fiber is deposited onto the surface of the collector.

The increase in tensile stress in Eq. (9) is chosen to eliminate various deficiencies. This change in volume flow rate is always accompanied by the transformation of the length, width and thickness at the top of the droplet in Fig. 1, showing the extreme difficulty in increasing the tensile stresses by controlling these factors. A combination of an increase in the linear velocity of the collector and a decrease in the deposition time of the fiber (Δ*t*) enables an effective increase in the tensile stress in Eq. (9) to eliminate various deficiencies.

In the deposition of jet fibers onto microstructures on the surface of the collector, the deposition position is mainly determined by the distribution of the electric field intensity on the surface of the microstructures^19^. According to the principle of potential superposition, the electric field intensity at any point in the *x*-*z* plane on the surface of the carbon micropillars in Fig. 1 is obtained as follows:

10 $${E}_{P}=\frac{\lambda }{2\pi {\varepsilon }_{0}\sqrt{2{{\rm{x}}}^{2}+\frac{{z}^{4}}{{x}^{2}}+3{z}^{2}}}\,(0\le x\le {L}_{C},\,0\le z\le {L}_{C})$$

where *E*_P_ is the electric field intensity, *λ* is the electric field line density between the carbon micropillars, *Ɛ*_0_ is the vacuum dielectric constant, and *L*_C_ is the radius of the carbon micropillars.

By analyzing Eq. (10), x = ±0.84*z* is found to be the critical condition for the maximum value of the electric field intensity on the surface of the carbon micropillars. The deposition position of the jet fiber deposited into the carbon micropillars is a result of *x* = ±0.84*z*.

## Results and discussion

### Nanoforming of PAN-based jet fiber during near-field electrospinning

**Fig. 2:**
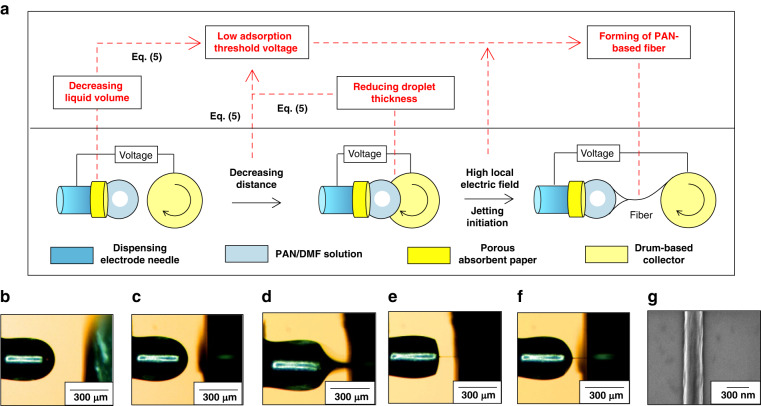
Electrostatic jet initiation during near-field electrospinning. **a** Schematic design of jet initiation. **b**–**f** Evolution from electrospinning solution to jet by decreasing the needle–drum distance in **b**, **c**, poking the rotating drum into droplets at the top of the dispensing needle in **d**, initiating jet at adsorption threshold voltage of 500 V in **e**, and forming continuous jetting in **f**. **g** Scanning electron microscopy image of a PAN-based fiber derived from a jet

The analysis is based on Eqs. (5) and (7) shows that the decreases in the thicknesses of droplets and the distance between the dispensing needle and the drum easily reduce the adsorption threshold voltage, thereby manufacturing thin polymer fibers. As shown in Fig. 2a, a porous absorbent paper is introduced and placed at the top of the dispensing electrode needle to absorb some of the liquid to reduce the thickness of the droplet. Further reduction in the droplet thickness is implemented as follows: a portion of the liquid in the droplet is adsorbed onto the surface of the drum by decreasing the distance between the dispensing needle and the drum, and is carried away by the drum, as described in Fig. 2a. Moreover, an artificial instability at the droplet–drum interface is introduced by this poking-in action to make a very high local electric field and to produce a large enough electrical stress to initiate jetting^16^. These actions allow the manufacturing design of thin pyrolyzable PAN-based polymer fibers, as shown in Fig. 2a.

The process of electrostatic jet initiation based on near-field electrospinning as described in Fig. 2a is designed by the above theoretical analysis, and it is implemented as shown in Fig. 2b–f. By using porous absorbent paper, decreasing the needle–drum distance (Fig. 2b, c), and poking the rotating drum into the droplets at the top of the dispensing needle (Fig. 2d), the volume of the droplet at the top of the needle tip is drastically reduced (Fig. 2e), enabling jetting initiation at a relatively low adsorption threshold voltage of 500 V (Fig. 2e, f). Under the conditions of jetting initiation, the jet moves toward the drum and is accompanied by the partial evaporation of solvent, forming PAN-based polymer fibers on the surface of the silicon wafer on the drum. These fibers are characterized using a scanning electron microscope, showing a diameter of ~240 nm (Fig. 2g). This indicates that a preliminary manufacturing design based on theoretical analysis can ensure continuous jetting at a low adsorption threshold voltage (500 V) and fabricate pyrolyzable PAN-based polymer fibers at the submicron scale.

**Fig. 3:**
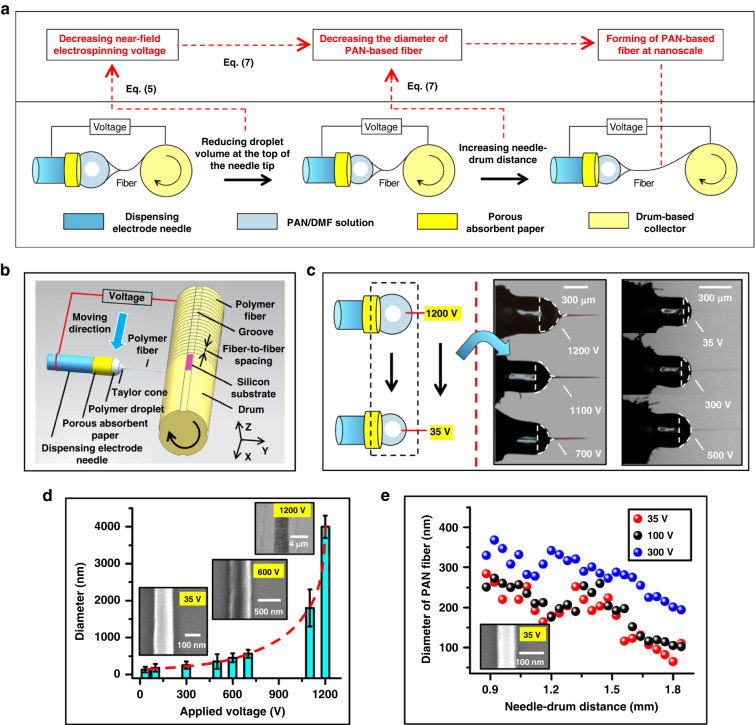
Forming of PAN fibers from the submicron scale to the nanoscale. **a** Nanomanufacturing design of PAN fibers based on submicro-jetting in Fig. 2. **b** Continuous deposition of PAN-based submicrofibers onto a silicon substrate on a rotating drum at an adsorption threshold voltage of 500 V. **c** Evolution of reducing the applied voltage by decreasing the volume of liquid at the top of the needle tip. **d** Diameter of the PAN-based jet fiber as a function of the applied voltage. **e** Dependence of the diameter of the PAN fiber on the needle–drum distance

By theoretically analyzing Eq. (5) and Eq. (7), the submicron-to-nanoscale process of forming PAN-based fiber is designed as described in Fig. 3a. In accordance with this design, using electrostatic jet initiation in near-field electrospinning, PAN-based jet fibers at the submicron scale are continuously deposited on the surfaces of the silicon substrates on the grooves of the drum by moving the dispensing electrode needle along the x-axis in Fig. 3b, forming an array of PAN-based fibers. During the deposition of the jet-based fiber, experimental investigation into the effect of the electrospinning liquid volume on the minimum voltage reveals a decreasing trend of voltage versus liquid volume, as shown in Fig. 3c. This highlights the possibility of reducing the voltage from 1200 V to 35 V by decreasing the electrospinning liquid volume. Jet fibers with various voltages are characterized by a scanning electron microscopy, and the corresponding diameter dimensions are obtained as shown in Fig. 3d. The effect of voltage on the diameter of the jet fiber in Fig. 3d shows that the average diameter of the jet fiber decreases to ~120 nm with the applied voltage. The dependence of the diameter on the electrospinning liquid volume at the top of the droplet and the voltage applied to the needle and drum are consistent with the results derived from the analysis of Eq. (7), providing a possibility for the preparation of PAN fibers with sizes close to the nanoscale.

On the basis of this dependence, the control of the needle–drum distance is introduced to further reduce the fiber diameter according to the analysis of Eq. (7). Further investigation into the effect of the needle–drum distance on the diameter of PAN fiber reveals a decreasing trend of the diameter versus the needle–drum distance at various voltages in Fig. 3e, including 35 V, 100 V and 300 V. An increase in the needle–drum distance to 1.8 mm is accompanied by a decrease in the diameter of the PAN fiber to ~80 nm, achieving the nanoformation of PAN-based jet fibers during near-field electrospinning.

The nanoforming process in near-field electrospinning demonstrates the advantages of fabricating pyrolyzable PAN-based jet fibers at the nanoscale using mathematical models of diameter and tensile stress. This model overcomes the difficulty in reducing the diameter of PAN-based fibers to the nanoscale for thin carbon fibers fabricated by the stabilization and pyrolysis of PAN-based fibers. The processing designs of poking the drum into the droplet at the top of the needle tip during electrostatic jet initiation, reducing the liquid volume at the tip of the needle during continuous jetting, and decreasing the needle–drum distance exhibit the key of the nanoforming process to fabricate PAN-based fibers at the nanoscale.

### Nanodeposition of PAN-based jet fiber in near-field electrospinning

**Fig. 4:**
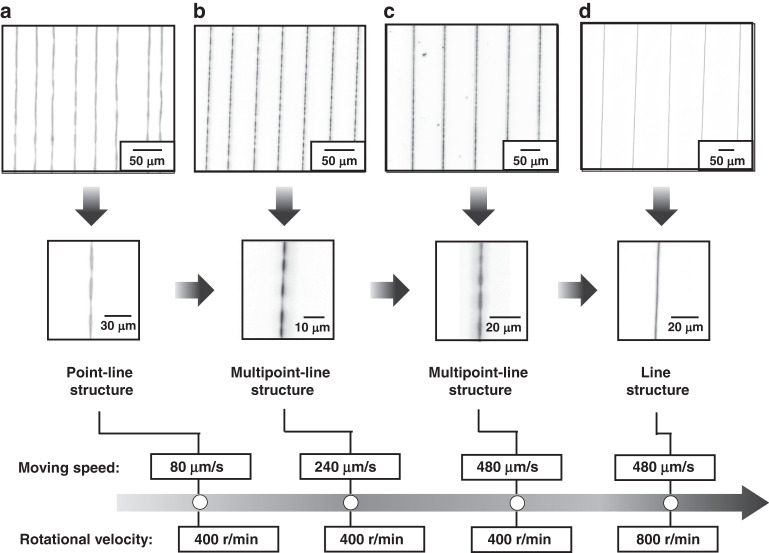
Transition from the point–line structure to line structure in the array of PAN-based jet fibers. **a**–**c** Arrays of PAN fiber formed at moving speeds of 80 μm/s, 240 μm/s, and 480 μm/s, respectively, at a rotational velocity of 400 r/min. **d** Straight alignment pattern of line-structure-based fibers at a moving speed of 480 μm/s and a rotational velocity of 800 r/min

During the deposition of jet-based fiber, a rotating drum is introduced into low-voltage near-field electrospinning to increase the tensile stress with an increase in the linear velocity of the collector and a decrease in the deposition time of the fiber (Δ*t*), as shown in Eq. (9), to eliminate various deficiencies. At a rotational velocity of 400 r/min for the rotating drum, a PAN-based fiber array is formed on the surface of the silicon substrate in Fig. 4a–d, and the point–line structure in Fig. 4a emerges as a result of a moving speed of 80 μm/s for the dispensing electrode needle. An increase in the moving speed from 80 μm/s to 240 μm/s allows the transition from a point–line structure to a multipoint–line structure in Fig. 4a, b as a result of the increase in the tensile stress of the jet fiber. The multipoint–line structures remain unchanged in Fig. 4b, c, even though the tensile stress of the jet fiber is increased to a relatively great extent by increasing the moving velocity from 240 μm/s to 2400 μm/s. Instead, the increase in the rotational velocity of the drum from 400 r/min to at least 800 r/min in Fig. 4c, d enables the transition from a multipoint–line structure to a line structure, eliminating various deficiencies arising from the point–line structure and multipoint–line structure. The effect of rotational velocity on tensile stress, as seen in Eq. (9), demonstrates the reason for the elimination of various deficiencies.

**Fig. 5:**
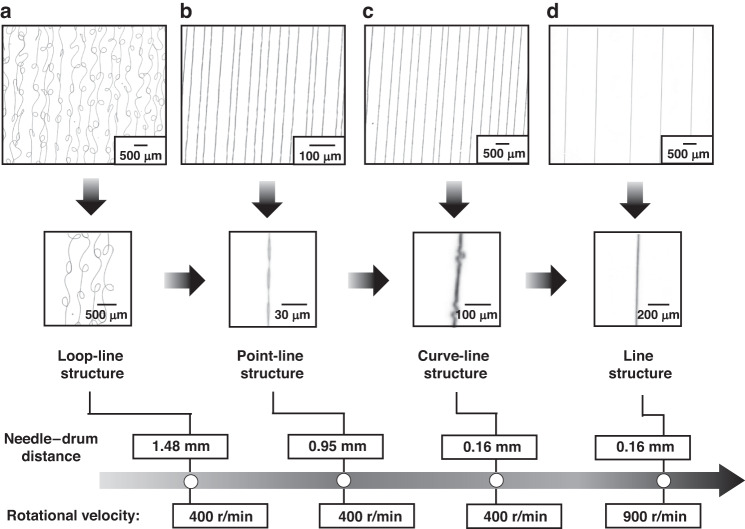
Transition from the loop–line structure to line structure in the array of PAN-based jet fibers. **a**–**c** Arrays of PAN fibers formed at needle–drum distances of 1.48 mm, 0.95 mm, and 0.16 mm, respectively, at a rotational velocity of 400 r/min. **d** Straight alignment pattern of line-structure-based fibers at a needle–drum distance of 0.16 mm and a rotational velocity of 900 r/min

A large needle–drum distance facilitates the nanoforming of jet fibers but is accompanied by a loop–line structure in Fig. 5a. Upon the reduction in the needle–drum distance from 1.48 mm to 0.16 mm, the loop–line structure is first converted to a point–line structure and then to a curve–line structure, as shown in Fig. 5a–c, by increasing the tensile stress, as shown in Eq. (9). Considering that a further reduction in the needle–drum distance to overcome the bending of the fibers in the curve–line structure cannot be implemented for avoiding the collision of the needle with the drum, the curve–line structure becomes the main deficiency in the array of jet fibers. In this case, an increase in the rotational velocity from 400 r/min to 900 r/min allows the transition from a curve–line structure to a line structure, showing an effective method for overcoming various deficiencies by increasing the tensile stress in Eq. (9).

**Fig. 6:**
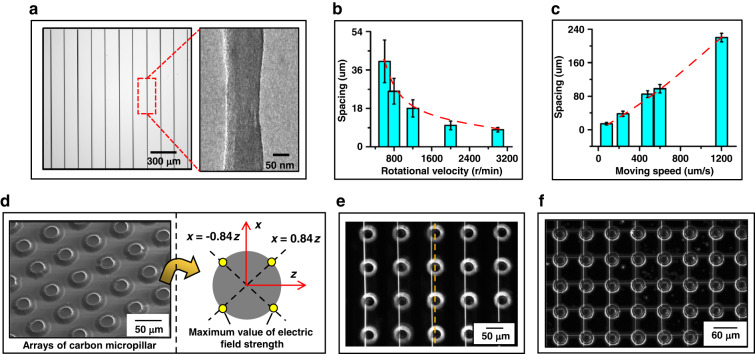
Micro/nanostructures based on PAN nanofibers and 3D carbon micropillars. **a** Array of PAN nanofibers deposited on the surface of a silicon substrate on a drum. **b**, **c** Dependence of highly uniform spacings between polymer nanofibers in an array on rotational velocity and moving speed. **d** Distributions of the maximum electric field intensity on the surfaces of 3D carbon micropillar arrays. **e** Array of PAN nanofibers deposited on the surfaces of 3D carbon micropillar arrays. **f** Micro/nanostructures formed by the deposition of PAN fiber arrays onto the surfaces of 3D carbon micropillars

With the control of moving speed, rotational velocity and needle–drum distance, the linear movement of the needle along the x-axis in near-field electrospinning enables the alignment of PAN nanofibers without deficiencies, forming an array of PAN nanofibers on the surface of the silicon substrate, as shown in Fig. 6a. Highly uniform spacing between PAN nanofibers in an array can be decreased to ~8 μm by maximizing the rotational velocity of the drum and by minimizing the moving speed of the needle, as shown in Fig. 6b, c.

For the micro/nanostructure based on PAN nanofibers, arrays of 3DCMPs in Fig. 6d are fabricated by the pyrolysis of SU-8 structures patterned with a high aspect ratio, as described in the carbon-MEMS method^20^. The location of the maximum electric field intensity on the surface of the 3DCMP is plotted based on the calculation of Eq. (10), thus obtaining the distribution of the maximum electric field intensity, as shown in Fig. 6d. The electric field strength directs the jet fibers to be deposited at the location with the maximum electric field strength, thus making an array of PAN nanofibers on the surfaces of 3DCMPs on the silicon substrate, as shown in Fig. 6e. With the 90-degree rotation of the silicon substrate with the structure of PAN nanofibers and 3DCMPs in Fig. 6e, an array of jet nanofibers is deposited onto the surfaces of 3DCMPs on this silicon substrate, forming the micro/nanostructure based on PAN nanofibers and 3DCMPs in Fig. 6f.

The nanodeposition of PAN-based jet fibers during near-field electrospinning shows the ability to fabricate PAN nanofiber arrays on 3DCMPs using a mathematical model of the electric field intensity on the surfaces of carbon micropillars. For 3DCMPs, the possibility of depositing and aligning PAN nanofibers on the surfaces and binding PAN nanofibers with the surfaces emphasizes the advancement in nanodeposition design. This allows the transition from PAN fibers to carbon fibers to occur at specific locations on the surfaces of 3DCMPs, allowing for the formation of carbon nanofiber structures on the 3DCMPs.

### Pyrolysis of PAN-based jet fiber for sub-10 nm carbon fiber arrays

**Fig. 7:**
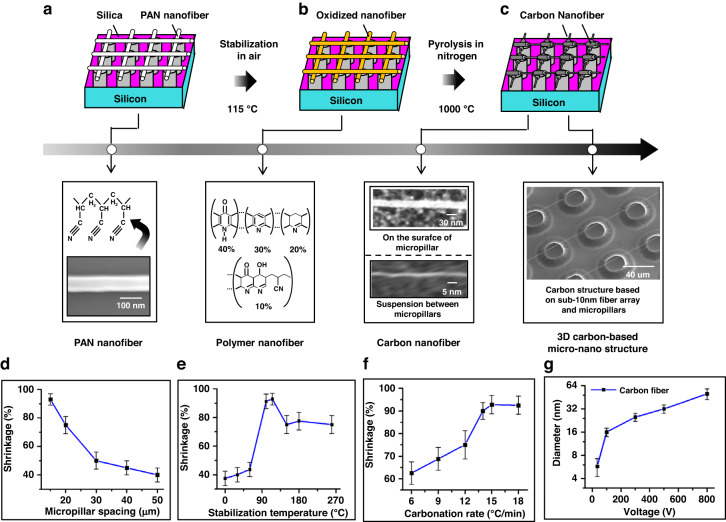
Micro/nanocarbon structures consisting of arrays of carbon nanofibers and 3D carbon micropillars. **a**–**c** Formation of carbon-based micro/nanostructures by the transformation of PAN fibers into carbon fibers using stabilization at 115 °C in air and pyrolysis at 1000 °C in nitrogen. **d**–**f** Dependence of the shrinkage from PAN-based fiber to carbon fiber on micropillar spacing, stabilization temperature, and carbonation rate, respectively. **g** Correlation between the diameter of the carbon fiber suspended between carbon micropillars and the voltage applied to the near-field electrospinning

The conversion of PAN-based jet fibers to carbon fibers is usually dependent on stabilization in air at 200–300 °C and subsequent carbonization in an inert atmosphere at 1000–1500 °C; it is accompanied by the submicroforming of carbon fibers with diameters ranging from 150 nm to 500 nm^21^. The micro/nanostructures based on PAN nanofibers and 3D carbon micropillars in Fig. 7a are placed in a furnace for this conversion; they are treated with stabilization at 115 °C in air and pyrolysis at 1000 °C in nitrogen, as shown in Fig. 7a–c. During stabilization, a ladder structure consisting of an acridone ring (40%), a naphthyridine ring (30%) and a hydro naphthyridine ring (20%), as shown in Fig. 7b, is formed as a result of cyclization and partial dehydrogenation, allowing the polymer fiber to withstand high temperatures during pyrolysis. In the following pyrolysis process, the polymer fiber is converted into carbon fiber with a diameter of ~4 nm due to the transition from ladder structure to carbon structure, as shown in Fig. 7c.

The fabrication of micro/nanostructures consisting of arrays of carbon nanofibers and 3D carbon micropillars relies on a protocol and a control method, enabling the diameter of carbon fibers suspended between 3D carbon micropillars to be reduced to less than 10 nm. The protocol consists of the following two parts: (1) PAN nanofibers suspended between carbon micropillars by deposition in near-field electrospinning to weaken the restriction effect of the micropillars on the radial contraction of the fibers during stabilization and pyrolysis and (2) the bond between the fibers and the surface of the carbon micropillar limiting the axial contraction of the suspended fibers. These enable the large stretching effect of the tension stress generated by stabilization and pyrolysis on the suspended fiber. The resulting diameter (25 nm in Fig. 7c) of the carbon nanofibers on the surface of the carbon micropillars is larger than that (4 nm in Fig. 7c) of the carbon nanofibers suspended between the carbon micropillars, demonstrating the importance of this protocol for the formation of arrays of carbon nanofibers with diameters of less than 10 nm.

Based on this protocol, the control method referring to the shrinkage and diameter characteristics is introduced by further investigation into the effects of micropillar spacing, stabilization temperature and carbonation rate on shrinkage during stabilization and pyrolysis and into the correlation between the carbon fiber diameter and voltage, as shown in Fig. 7d–g. A micropillar spacing of 15 μm, a stabilization temperature of 15 °C, and a carbonation rate of 15 °C/min are selected for the shrinkage of fiber to 96%, showing the control method for increasing the shrinkage of fibers during the conversion of PAN fibers to carbon fibers. In combination with the high shrinkage of fibers, the reduction in voltage to 35 V allows this control method to decrease the diameter of the carbon fiber to less than 10 nm, obtaining sub-10 nm carbon fiber arrays on 3D carbon micropillars. The fabrication of sub-10 nm carbon fiber arrays on 3D carbon micropillars highlights the advancement in the manufacturing design of 3D micro/nanocarbon structures by nanoforming, nanodeposition and pyrolysis methods using these established mathematical models of diameter and tensile stress for jet fibers and the electric field intensities on the surfaces of carbon micropillars.

## Conclusion

In this work, we emphasize that sub-10 nm carbon fiber arrays on 3D carbon micropillars can be fabricated by a technique consisting of nanoforming and pyrolysis of PAN-based jet fibers. This technique shows the ability to reduce the diameter of PAN-based jet fibers to ~80 nm by the use of nanoforming and nanodeposition methods, providing an opportunity for the nanofabrication of pyrolyzable polymer fibers. Moreover, the ability of this technique to deposit nanoscale PAN-based jet fibers onto semiconducting wafers, such as silicon and 3D carbon micropillars, in an ordered arrangement enables the direct incorporation of strongly correlated properties in conventional semiconductors during pyrolysis, allowing for a new generation of multifunctional electronic devices. These advantages suggest that this technique can produce a 3D micro/nanocarbon structure based on nanofibers and micropillars, greatly expanding the range of available 3D carbon microstructures. The sub-10 nm size of carbon fibers offer further perspective on enhancing the scale-to-property effects^13,22^ of 3D micro/nanocarbon structures in potential applications, such as high-rate supercapacitors^23^, absorption-dominated electromagnetic interference shields^24^, capacitive deionization devices^25^, and electrocatalytic performance enhancers^26^.
